# Thyroid peroxidase (TPO) expressed in thyroid and breast tissues shows similar antigenic properties

**DOI:** 10.1371/journal.pone.0179066

**Published:** 2017-06-02

**Authors:** Marlena Godlewska, Katarzyna D. Arczewska, Magdalena Rudzińska, Anna Łyczkowska, Wanda Krasuska, Karolina Hanusek, Jean Ruf, Mirosław Kiedrowski, Barbara Czarnocka

**Affiliations:** 1 Department of Biochemistry and Molecular Biology, Center of Postgraduate Medical Education, Warsaw, Poland; 2 UMR-MD2, Aix-Marseille University, Marseille Medical School, Marseille, France; 3 Clinical Department of Oncology and Hematology, Central Clinical Hospital of the Ministry of Interior in Warsaw, Warsaw, Poland; Baylor College of Medicine, UNITED STATES

## Abstract

**Background:**

Thyroid peroxidase (TPO) is essential for physiological function of the thyroid gland. The high prevalence of thyroid peroxidase antibodies (TPOAbs) in patients with breast cancer and their protective role had previously been demonstrated, indicating a link between breast cancer and thyroid autoimmunity. Recently, TPO was shown to be present in breast cancer tissue samples but its antigenicity has not been analyzed.

**Methods:**

In this study, we investigated TPO expression levels in a series of fifty-six breast cancer samples paired with normal (peri-tumoral) tissue and its antigenic activity using a panel of well-characterized murine anti-human TPOAbs.

**Results:**

We have shown that TPO transcripts were present in both normal and cancer tissue samples, although the amounts in the latter were reduced. Additionally, we observed that TPO levels are lower in more advanced cancers. TPO protein expression was confirmed in all tissue samples, both normal and cancerous. We also found that the antigenicity of the immunodominant regions (IDRs) in breast TPO resembles that of thyroid TPO, which is crucial for effective interactions with human TPOAbs.

**Conclusions:**

Expression of TPO in breast cancer together with its antigenic activity may have beneficial effects in TPOAb-positive breast cancer patients. However, further studies are needed to confirm the beneficial role of TPOAbs and to better understand the underlying mechanism.

## Introduction

**Fig 1:**
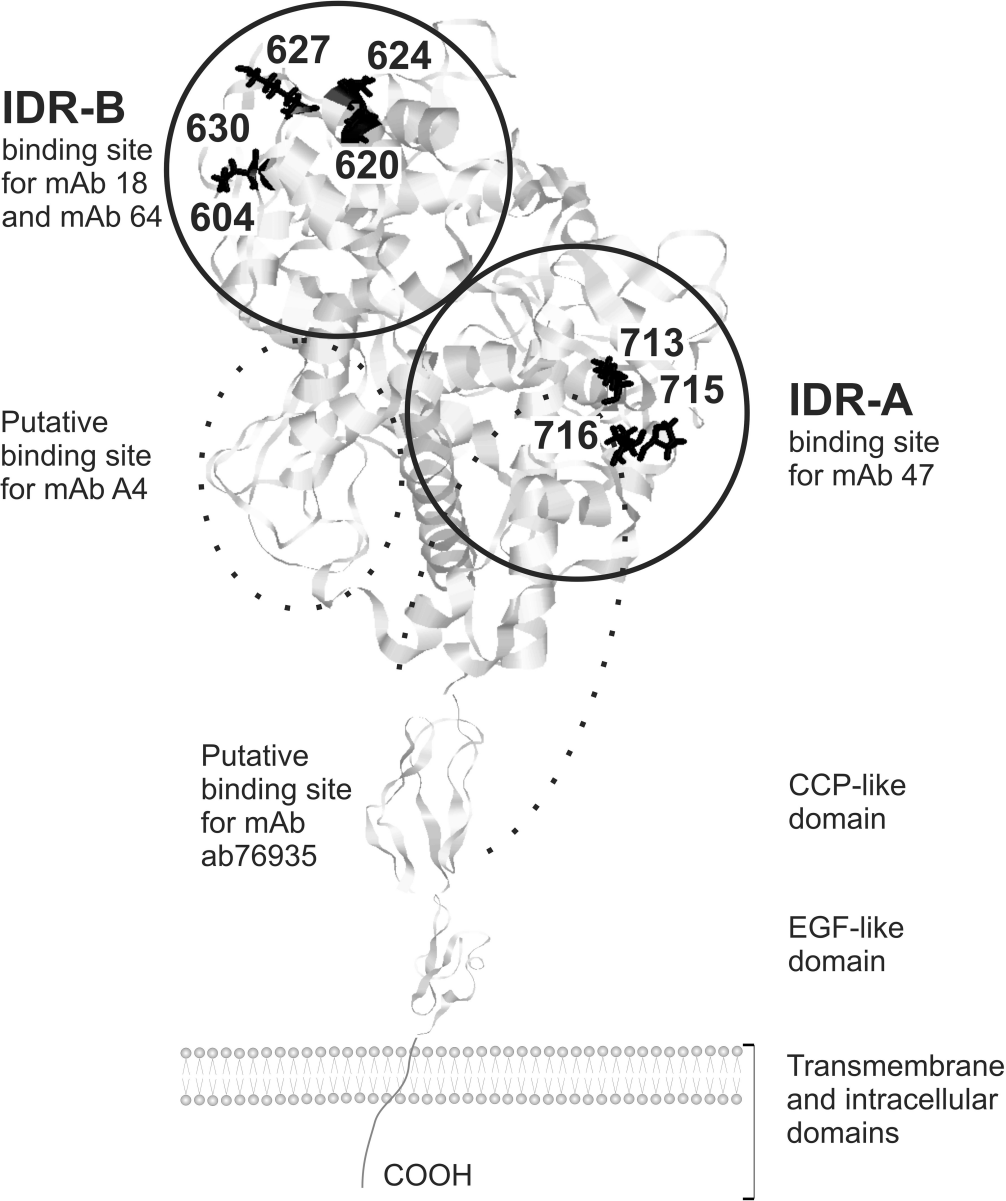
Predicted three-dimensional structure of the human thyroid peroxidase (TPO) protein. Localization of immunodominant region A (IDR-A) and B (IDR-B) with assumed binding sites for anti-TPO monoclonal antibodies used in the study is shown. Key contact amino acid residues within IDR-A and -B domains which are involved in the binding of these antibodies are shown in black and annotated [6, 7]. The model was adjusted using the Swiss-PDB Viewer 4.1 available at http://www.expasy.org/spdbv/.

Thyroid peroxidase (TPO) belongs to the family of human peroxidases together with lactoperoxidase (LPO), myeloperoxidase (MPO) and eosinophil peroxidase (EPO). Its key physiological function is biosynthesis of thyroid hormones. The human *TPO* gene encodes a 933-amino acid protein with a molecular weight of approximately 100 kDa. According to a computer model, the mature TPO protein consists of a dominant ectodomain, in which MPO-like, complement control protein (CCP)-like and epidermal growth factor (EGF)-like domains can be identified, followed by short transmembrane and cytoplasmic regions (Fig 1) [1, 2]. During intracellular trafficking to the cell membrane, TPO undergoes several post-translational modifications such as proteolytic trimming, glycosylation, heme fixation, and dimerization (reviewed in [3–5]).

The association between thyroid diseases, especially thyroid autoimmunity, and breast cancer has been widely studied. Several studies demonstrated that the levels of thyroid peroxidase autoantibodies (TPOAbs) are increased in breast carcinoma patients [8–13]. Furthermore, the presence of TPOAbs was shown to represent a protective factor in these patients [11, 14] but this finding remains controversial [12]. Additionally, the presence of TPOAbs was associated with a reduced incidence of distant metastases in breast cancer patients [15]. Moreover, in a very recent study, Brandt and collaborators [16] found positive association between free thyroxin (T4) and improved survival following breast cancer but their analyses also indicated that the TPOAbs status may affect survival: they observed a tendency towards an increased survival in TPOAbs-positive patients.

TPOAbs are a hallmark of autoimmune thyroid disease (AITD) (reviewed in [4, 5, 17]). In the first study on the reactivity of TPOAbs with TPO epitopes, Ruf and collaborators used competition assays and murine monoclonal anti-TPOAbs and TPOAbs from patients with AITD, and showed that the autoimmune response to TPO is predominantly dependent on two partly overlapping immunodominant regions (IDRs) on the peroxidase surface: IDR-A and IDR-B [18]. Further studies using recombinant Fab fragments-TPOAbs fully confirmed these findings, although reversed nomenclature of the immunodominant region was used [19, 20]. In this paper, we apply the nomenclature originally proposed by Ruf and collaborators [18].

Although the expression of TPO in breast cancer has long been postulated, the presence of TPO in breast cancer tissue has only recently been shown [21]. Full-length TPO1 isoform was detected, together with several shorter TPO mRNA transcripts. In this study, we aimed to determine whether TPO expressed in breast tissue is immunologically similar to that expressed in the thyroid. To this effect, we have investigated TPO expression levels in fifty-six breast cancer samples and in paired adjacent normal tissue using a panel of well-characterized murine anti-human TPOAbs and human TPOAbs.

## Materials and methods

### Patients and samples

Fifty-six surgical primary tumor samples paired with the adjacent non-cancerous tissues were obtained from patients with breast cancer of different stages who were hospitalized at the Maria Skłodowska-Curie Memorial Cancer Center and Institute of Oncology (Warsaw, Poland). Tissue specimens were snap-frozen in liquid nitrogen and kept at -70°C before RNA and/or protein extraction. Formalin-fixed paraffin-embedded tissues obtained from the same patients were used for immunohistochemical analysis. Archived normal and Graves’ disease thyroid tissue samples were used as controls. The study and all experimental procedures were approved by the local Ethical Committee at the Memorial Cancer Center and Institute of Oncology and at the Center of Postgraduate Medical Education (Warsaw, Poland) and the informed written consent was obtained from all patients.

### RNA extraction and real-time quantitative PCR (RT-qPCR)

Total RNA was isolated from breast tissue samples using Tissue Lyser (Qiagen, Hilden, Germany) and RNeasy Lipid Tissue Mini Kit (Qiagen) and the on-column DNase digestion with RNase-free DNase Set (Qiagen) following the manufacturers’ instructions. Total RNA (500 ng) was reverse-transcribed using the High Capacity cDNA Reverse Transcription Kit with RNase Inhibitor (Thermo Fisher Scientific, Rockford, IL, USA) according to the recommended protocol. The expression of TPO and 18S rRNA was quantified by RT-qPCR using Maxima SYBR Green/Fluorescein qPCR Master Mix (Fermentas, Glen Burnie, MD, USA), cDNA, and 0.5 μM of specific oligonucleotide primers (listed below):

TPO (NM_000547):
  Forward: 5’-GCTTGCAACAACAGAGACCAC-3’,
  Reverse: 5’-CTGAAGCCGTCCTCATAGACTG-3’;
18S rRNA (NM_02255):
  Forward: 5’-CCAGTAAGTGCGGGTCATAAG-3’,
  Reverse: 5’-CCATCCAATCGGTAGTAGCG-3’.

RT-qPCR was performed in IQ5 Realtime PCR (Bio-Rad, Munich, Germany) with 1 cycle of 10 min at 95°C followed by 40 or 50 cycles of 15 s at 95°C, 30 s at 55°C and 30 s at 72°C for 18s rRNA and TPO mRNA analysis, respectively. For each sample, the expression of the target gene was normalized using the levels of 18S rRNA. For a standard curve, a series of dilutions of a cDNA pool obtained from human thyroid tissues was used. Two cDNA preparations per specimen were synthesized and analyzed independently in triplicates.

### Antibodies

Following TPO-specific antibodies were used: mouse monoclonal antibodies (mAbs) 47, 18, 64 [18], a rabbit antiserum to peptide P14 (aa 599–617) [1, 22, 23], mAb A4, that was kindly provided by Prof. J. P. Banga (King’s College London, UK) [24], and mAb ab76935 purchased from Abcam (Cambridge, UK). Rabbit polyclonal antibody 10376-1-AP specific to human LPO, murine isotype IgG1 control, and β-actin-specific mAb (clone AC-15) was obtained from Proteintech (Chicago, IL, USA), Dako (Glostrup, Denmark), and Abcam, respectively. Healthy donor sera (n = 5) and AITD patient sera (n = 5) were used to prepare TPOAbs negative and positive serum pools. Preimmune rabbit IgG were isolated from rabbit sera.

### Protein extraction, thyroid peroxidase (TPO) immunoprecipitation and Western blotting

Tissue samples were homogenized using Tissue Lyser (Qiagen) in ice-cold RIPA buffer (Thermo Fisher Scientific) supplemented in Complete Protease Inhibitor Cocktail (Roche, Mannheim, Germany). Protein concentration was determined by the BCA Protein Assay Kit (Thermo Fisher Scientific), then the protein extracts were aliquoted and stored at -70°C until analysis.

Breast tissue lysates (200 μg) were incubated with rabbit anti-peptide P14 serum overnight at 4°C, before capturing the immune complexes by a 2-hour incubation with protein A-Agarose (Merck Millipore, Darmstadt, Germany) at room temperature. The agarose beads were then collected, washed five times in phosphate-buffered saline (pH 7.3), and the immunoprecipitated proteins were finally analyzed by SDS-PAGE and Western blotting.

Primary antibodies were preabsorbed using the excess of highly purified human TPO (hTPO) isolated from the thyroid gland as already described [25] or with bovine LPO (bLPO; Sigma-Aldrich, Steinheim, Germany). Briefly, a piece of nitrocellulose (2 × 2 cm) was saturated with hTPO or bLPO for one hour and blocked in 5% skimmed milk (w/v) in Tris-buffered saline (TBS)-0.1% Tween 20 (v/v) for one hour, then the appropriately diluted primary antibody (Ab) was added and samples were further incubated for three hours. Control reactions without the antigen on the membranes were performed for each primary antibody under the same assay conditions. All steps were performed at room temperature.

Standard Western blot analyses were performed using primary antibodies at a concentration of 1 μg/ml (anti-human TPO mouse monoclonal antibodies (mAbs)) or 33 ng/ml (anti-human LPO 10376-1-AP Ab), followed by an incubation with the secondary HRP-conjugated rabbit anti-mouse IgG (Jackson ImmunoResearch, West Grove, PA, USA) or HRP-conjugated goat anti-rabbit IgG (Dako, Glostrup, Denmark). Signals from reactive bands were visualized with SuperSignal West Pico Chemiluminescent Substrate or SuperSignal West Dura Extended Duration Substrate (Thermo Fisher Scientific). Protein bands were analyzed using the GelAnalyzer 2010a software available at http://www.gelanalyzer.com.

### Immunohistochemistry and immunofluorescent staining

Tissue sections were prepared from the blocks of tumor tissue. Slices (3–4 μm) were dewaxed, deparaffinized, rehydrated, and pretreated for antigen retrieval with the target retrieval solution (TRS, pH 9.0, Dako) for 20 min at 98°C. After the quenching of endogenous peroxidase activity (0.3% H_2_O_2_ (v/v), 15 min), tissue sections were incubated overnight at 4°C in a humid chamber with murine monoclonal anti-TPO antibodies or isotype IgG control antibody (Dako) at 14 μg/ml in 10% goat serum (v/v). This was followed by the incubation with REAL EnVision Detection System (Dako) for 30 min at room temperature. Between steps, slices were intensively washed in TBS-1% Tween 20. Immunoreaction was visualized with 3′3′-diamino-benzidine (DAB; Dako). The sections were counterstained with hematoxylin, mounted, and examined under a light microscope (Olympus BX41, Japan). Thyroid tissue samples obtained from patients with Graves’ disease were used as positive controls. The results of the immunohistochemical staining were then evaluated by two independent experts and scored as negative or positive.

The immunofluorescence-stained sections were subsequently used for TPOAb binding analysis. Following the antigen retrieval and blocking of non-specific reactions with 10% mouse serum (v/v) in TBS-1% Tween 20, slices were incubated for one hour at room temperature with pooled human serum diluted 1:200. After intensive washing (TBS-1% Tween 20, four times, 5 min each, sections were incubated for one hour at room temperature with mouse anti-human IgG phycoerythrin (PE)-conjugate (BD Biosciences, San Jose, CA, USA). Following the washing, slices were stained with 4',6-diamidino-2-phenylindole, dihydrochloride (DAPI), mounted, and visualized using the LSM800 confocal microscope with ZEN 2.1 software (Zeiss, Göttingen, Germany).

### Statistical analysis

All experiments were performed at least four times unless stated otherwise. All quantitative data were expressed as means ± standard deviation (STDEV). The obtained RT-qPCR data were compared between tumor and normal breast tissue samples using Wilcoxon’s test, while the effects of cancer histological grade and stage of lymph node metastasis were tested using the Mann-Whitney U test. Immunohistochemistry results were evaluated with the χ^2^ test. *P*-values below 0.05 were considered as indicative of a statistical significance. *P*-values for trend were calculated using the Wilcoxon-type test for trend [26]. All statistical analyses were performed using the Statistica 12 software (StatSoft, Inc., USA).

## Results

In order to determine whether the expression of thyroid peroxidase and its antigenic activity in breast tissue are similar to those in the thyroid, we have analyzed its expression at both mRNA and protein level in breast cancer and normal breast tissue from fifty-six breast cancer patients compared to normal thyroid. Basic patient and tumor characteristics are listed in Table 1.

**Table 1:** Basic characteristics of patients and tumors.

	n (%)	
**Patients (n = 56)**		
Age at diagnosis (years)		
< 55	13 (23%)	
≥ 55	42 (75%)	
NA	1 (2%)	
Mean ± STDEV		64.9 ± 11.1; range: 43.0–86.0
Gender		
Male	1 (2%)	
Female	55 (98%)	
**Tumors (n = 56)**		
Histological type		
invasive breast carcinoma, hhnon-specific type (NST)	45 (80%)	
breast cancer, other types	11 (20%)	
Tumor size (cm)		
< 2 cm	13 (23%)	
≥ 2 cm	42 (75%)	
NA	1 (2%)	
Mean ± STDEV		2.8 ± 2.1; range: 0.5–11.0
Histological grade		
G1	9 (16%)	
G2	21 (38%)	
G3	20 (36%)	
NA	6 (10%)	
T stage		
T1	20 (36%)	
T2	22 (39%)	
T3/T4	9 (16%)	
NA	5 (9%)	
LN status		
LN-negative	28 (50%)	
LN-positive	23 (41%)	
NA	5 (9%)	

STDEV, standard deviation; NA, data not available; LN-positive, local metastasis to lymph nodes; LN-negative, no local metastasis to lymph nodes.

### Thyroid peroxidase (TPO) mRNA expression in human breast tissue

**Fig 2:**
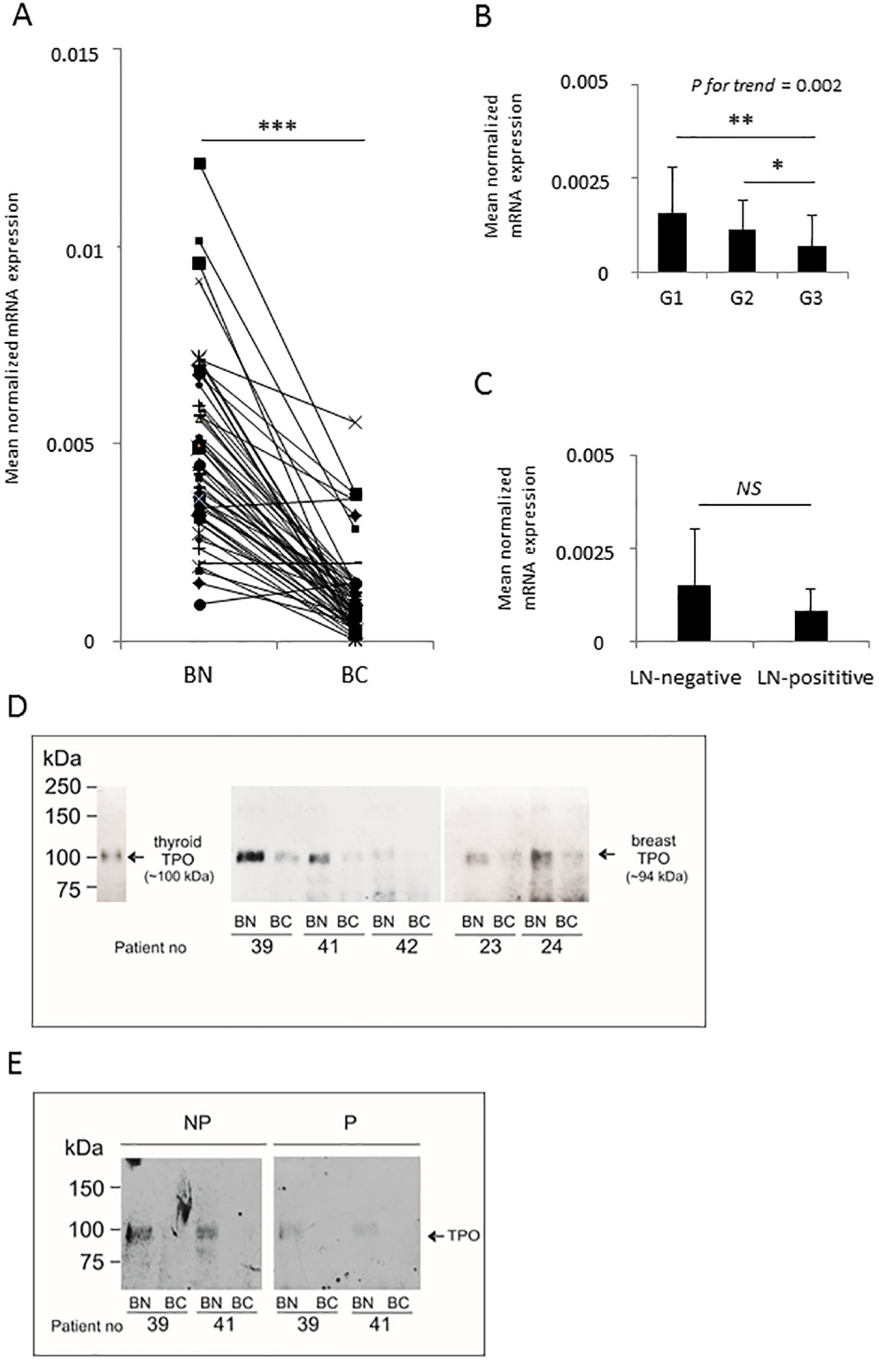
Thyroid peroxidase (TPO) expression in normal and cancerous breast tissue samples in fifty-six patients. (A) RT-qPCR analysis, (B and C) RT-qPCR analysis of TPO mRNA expression in breast cancer samples according to the cancer grade (B) and the patient nodal status (C). (D-E) TPO protein expression in normal and cancerous breast tissue representative samples by Western blotting. (D) TPO was immunoprecipitated using anti-peptide P14 Ab, and detected with an anti-TPO monoclonal antibody: mAb 47. (E) ab76935 antibody preabsorbed with the excess of highly purified TPO (P) (right panel) or non-preabsorbed ab76935 (NP) (left panel) were used. *: *P-value* < 0.05; **: *P-value* < 0.01; ***: *P-value* < 0.001; NS: not significant; BN: peri-tumoral; BC: breast cancer tissue.

We analyzed tissue samples from fifty-six breast cancer patients. First, we evaluated the *TPO* gene expression in matched cancer and normal tissue using RT-qPCR with a pair of primers (forward: exon 5/6, reverse: exon 6) amplifying all known TPO isoforms. TPO mRNA expression was detected in all samples (Fig 2A). The expression level of TPO in normal tissues was on average at least four times higher than in the matched cancer samples and this difference was statistically significant (*P*-value < 0.001). Additionally, we observed statistically significant differences in TPO mRNA expression in association with the tumor grade (Fig 2B). TPO mRNA levels decreased with the tumor progression: in poorly differentiated (grade 3) tumors they were 1.6-fold lower than in moderately differentiated (grade 2) tumors (*P*-value < 0.05) and 2.3-fold lower than in well-differentiated (grade 1) tumors (*P*-value < 0.01), with the *P*-value for trend of 0.002. We have also analyzed a possible association of TPO levels and the lymph node status in breast cancer patients. Although a 1.8-fold decrease in TPO mRNA levels was observed in samples from patients with local metastases to lymph nodes as compared to patients without such metastases (Fig 2C), this difference was not statistically significant (*P*-value = 0.2).

### Thyroid peroxidase (TPO) protein expression in human breast tissue

All antibodies, both monoclonal and polyclonal, were initially tested on normal thyroid tissue. The staining confined to the apical membrane of the thyroid follicular cells was observed, confirming the specific recognition of TPO protein (data not shown). To investigate whether anti-TPOAbs cross-react with LPO, we analyzed the reactivity of anti-TPOAbs using LPO. In S1 Fig, Western blot results are presented, showing that all TPO-specific monoclonal antibodies recognizing linear epitopes (mAb 47, mAb A4, and ab76935) do not bind to bovine LPO. Furthermore, human LPO-specific antibody (10376-1-AP) does not recognize human TPO (see S1 Fig). We observed no cross-reactivity between TPO autoantibodies and bovine LPO in our pool of AITD patient sera, while human TPO, used as a positive control, was effectively immunoprecipitated with the same autoantibodies. As expected, no binding to bovine LPO and human LPO was observed when TPOAbs-free pooled serum was used (see S2 Fig).

We analyzed TPO protein expression in paired cancer and normal breast surgical samples from twelve breast cancer patients by immunoprecipitation with anti-peptide P14 serum followed by immunoblotting (Fig 2D). The average TPO protein expression level was 151 ± 134 ng and 115 ± 104 ng of TPO per 1 mg of crude tissue lysate. The difference in TPO protein expression between normal tissue and cancer samples was borderline significant (*P*-value = 0.05). A significant reduction in TPO levels was observed when using TPO-preabsorbed ab76935 antibody to verify the specificity of detected bands (Fig 2E). The molecular weight of detected breast tissue-expressed TPO was 94.0 ± 1.5 kDa, which is lower than that measured in the thyroid (about 100 kDa).

**Fig 3:**
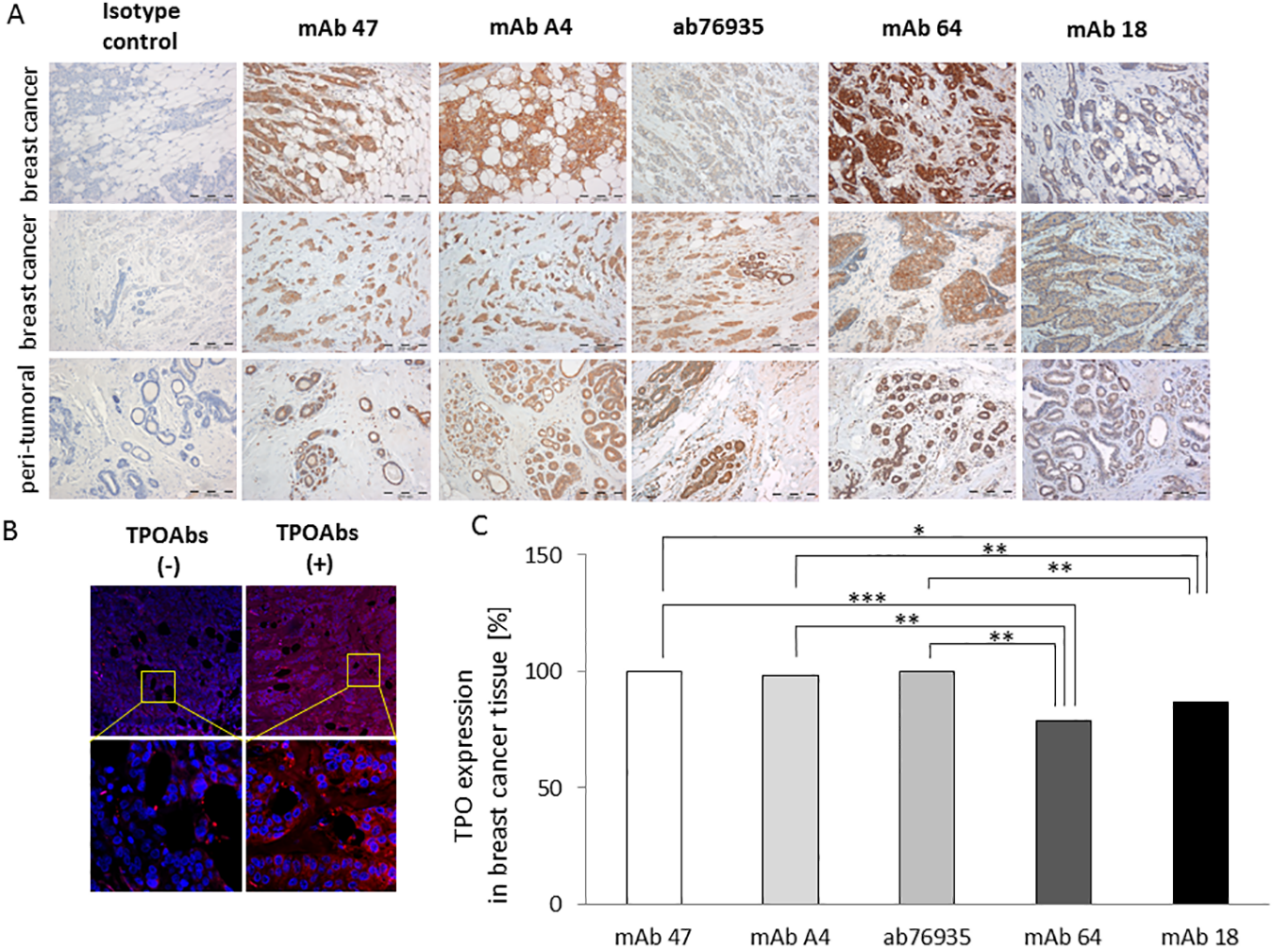
Expression of thyroid peroxidase (TPO) protein in breast tissue samples. Representative immunostaining results obtained with a panel of monoclonal antibodies (mAbs), (A) and human serum pools (B). Representative images for two breast cancer tissues and one peri-tumoral tissue are shown. Magnification, 100×. (B) Positive staining (red) observed in TPOAb-positive serum pool obtained from patients with autoimmune thyroid disease (AITD; TPOAbs(+)), whereas serum negative for TPOAbs (TPOAbs(-)), used as a negative control, gave weak or no staining. Nuclei (blue) were counterstained with DAPI. Magnification, 200× and 630×. (C) The frequency of TPO positive staining in breast cancer tissue samples (n = 56). In all cases, TPO protein was detected by mAb 47, mAb A4, and ab76935, while mAb 64 and 18 detected TPO in 79% and 87% of all cases, respectively. *: *P-value* < 0.05; **: *P-value* < 0.01; ***: *P-value* < 0.001.

Subsequently, the TPO protein expression was evaluated in samples from all fifty-six breast cancer patients by immunohistochemistry using a broad panel of TPO-specific monoclonal antibodies that recognize linear (mAb 47, mAb A4, and ab76935) or conformational (mAb 18 and mAb 64) epitopes of the TPO immunodominant region. Images presented in Fig 3A represent the range of the obtained results. TPO expression was detected in both cancer cells and in the peri-tumoral ductal epithelium (considered as normal breast tissue). Varying TPO staining intensities were observed in cancer cells. In nearly all examined breast cancer tissue samples, positive TPO immunostaining was observed when three monoclonal antibodies recognizing linear epitopes were used: mAb 47 (positive staining observed in 52 out of 52 exploitable samples), mAb A4 (50 out of 51) and ab76935 (52 out of 52). Matched surgical margins were available for 31 samples. Using the same monoclonal antibodies on this peri-tumoral (non-cancerous) tissue, we found TPO expression in 90% (28 of 31; mAb 47), 87% (27 of 31; mAb A4), and 97% (30 of 31; ab76935) of samples. We have also evaluated the reactivity of mAb 18 and mAb 64 which recognize conformation-dependent epitopes, using the same samples. We found that these antibodies bound to TPO in 79% (41 of 52; mAb 18) and 87% (45 of 52; mAb 64) of breast cancer samples, and in 59% (17 of 29; mAb 18) and 63% (19 of 30; mAb 64) of normal breast specimens samples. Statistical analysis revealed that monoclonal antibodies specific to linear epitopes did not differ in breast cancer section staining intensities (Fig 3C). However, a statistically significant decrease in TPO staining intensity was observed when conformation-dependent antibodies were used, in comparison with conformation-independent (linear) antibodies (Fig 3C). No signals were obtained when isotype control staining was performed, confirming the specificity of the reaction.

TPO immunostaining was observed in normal and cancer breast tissue samples as well when pooled human serum with high levels of TPOAbs was used (Fig 3B). TPO expression was detected in breast cancer cells and in the adjacent peri-tumoral margins. However, the number of immunostained sections was too low to perform statistical analysis. The specificity of the staining reactions was confirmed by using pooled human TPOAbs-free serum as a negative control.

## Discussion

Thyroid peroxidase plays a key role in the biosynthesis of thyroid hormones by catalyzing oxidation of the inorganic iodide and coupling of iodinated tyrosine to form the thyroxin and triiodothyronine. Hence, it is essential for physiological function of the thyroid gland [4]. The TPO transcripts detected in normal thyroid gland encompass not only TPO1 but also several shorter spliced forms [27, 28]. Similarly, in malignant thyroid cancer many different spliced forms of TPO are detected, however total TPO transcript expression is lower than in normal thyroid gland and it is not associated with the tumor clinical stage [29, 30]. TPO is also one of the major thyroid antigens in autoimmune thyroid disease [31]. The majority of AITD patients are positive for TPO auto-antibodies. Although the physiological function of TPOAbs is not fully elucidated, there are reports evidencing their involvement in the thyroid gland destruction [32].

A recent study demonstrated that thyroid peroxidase mRNA and protein are also expressed in breast cancer tissue and in peri-tumoral regions [21]. In our study, we focused on the structure and immunological activity of breast TPO, since TPO autoantibodies are predominantly specific for the tertiary structure-dependent conformational epitopes [33, 34]. We detected lower expression levels of TPO in all breast cancer tissue samples than in matched normal tissue (surgical margins), even though it was previously demonstrated that TPO expression levels are similar in both of these tissues [21]. This discrepancy may result from a much higher number of samples analyzed in our study. We have observed a decrease in TPO expression in cancer tissue samples compared to non-cancerous tissue in all but three patients. Even though different primer pairs were used in our study and the previous one [21], both of these pairs should be able to recognize all known TPO isoforms. Additionally, we observed statistically significant decrease in the TPO expression levels in more clinically advanced breast cancer cases, and a decrease in TPO expression in patients with regional lymph node metastasis. However, this difference was not statistically significant. These data suggest that expression level of TPO mRNA could be associated with aggressiveness of breast cancer, although we have not found such a relationship in patients with different lymph node status. On the other hand, we have not clearly seen such a tendency on the protein level since the estimated amount of TPO protein measured in five G2 and five G3 tissue samples was similar, irrespectively of the breast tumor stage. Moreover, we did not find such an association using immunohistochemistry. In thyroid cancer, TPO protein expression was lower than in paired malignant normal tissues [35]. The fact that we did not observe such an association in normal mammary tissue from the same patients may result from a relatively small number of analyzed samples (12 cases) which was due to lack of sufficient amount of material (not enough material remained after RNA extraction). Enlarging the study population may reveal such a tendency also on the TPO protein level.

Approximately 25% of all TPOAbs in the sera of patients with thyroid autoimmune disorders are directed against TPO immunodominant region A (IDR-A) and 50% against IDR-B [36]. Therefore, we used several antibodies specific for IDR-A and IDR–B to investigate breast TPO immunoreactivity. The epitopes for mAb 18 and mAb 64 are located in region B [18]. We had previously performed a precise mapping of TPO amino acids involved in the binding of these conformation-dependent monoclonal antibodies. This study revealed that the epitopes for these two antibodies overlap, however, the key resides differ [7]. Breast TPO was also probed with mAb 47 which is representative of IDR-A-specific antibodies. Importantly, unlike in the above mentioned IDR-B-specific monoclonal antibodies, the mAb 47 epitope is insensitive to denaturation and reduction, and therefore it recognizes the native (properly fold) TPO as well as its partly or completely denatured form with the same efficiency [18]. Similarly, the mAb A4 epitope, located at the N-terminus, outside of IDR-A and -B, is denaturation-resistant. The precise localization of the ab76935 epitope is unknown and may be narrowed to the C-terminal region of the MPO-like domain and most of the CCP-like domain since the antibody was generated using a peptide corresponding to residues 672–780 of human TPO. Therefore, the ab76935 binding site is located in immunorective region of TPO, that is mainly restricted to MPO- and CCP-like domains (reviewed in [4, 5, 17]). Here we detected the positive TPO staining in all cancer and normal breast tissue samples. The reduction in the staining intensity we observed when structure-sensitive antibodies were used may be explained by minor changes in the tertiary structure of breast TPO. These modifications of the three-dimensional architecture may result from proteolytic cleavage occurring after surgical excision but before specimen fixation. Additionally, harsh conditions such as treatment with organic solvents or high temperature may have a harmful impact on the TPO conformation. We cannot exclude that the observed differences may be a result of the alternative splicing either. Many TPO isoforms were detected in breast cancer samples [21] and an increased proportion of TPO alternatively-spliced mRNA variants can be observed in various types of thyroid cancer [29]. The three-dimensional structure of some TPO protein isoforms may differ from that of the full-length TPO [28], and as a consequence the structure of their conformational epitopes may be impaired. Therefore, we could speculate that some proportion of TPO protein variants expressed in breast tissue is not recognized by conformation-sensitive antibodies, however, they are still immunoreactive for antibodies binding to linear epitopes. Even if spliced forms of TPO were present in the examined breast tissue samples, our results suggest that the proportion of immunologically active TPO constitute a dominant part of total breast TPO. Similar results were obtained in studies comparing TPO expression in thyroid cancer samples with that in paired normal thyroid tissue specimens [35].

Lactating breast and other glandular tissues such as salivary, lacrimal, tracheal, and bronchial glands express human lactoperoxidase (LPO) [37, 38]. The sequence homology between human LPO and TPO is as high as 46% [38]. Therefore, in the present study we considered the potential influence of LPO detection on the obtained results. All used murine antibodies do not recognize LPO, except mAb 64, which can slightly react with bLPO [18]. However, the significance of this cross-reactivity between mAb 64 and breast TPO/LPO is low, as shown by the similar reactivity patterns of mAb 64 and mAb 18 in the immunohistochemistry assay. Moreover, we observed staining using mAb 64 in peri-tumoral breast tissue in which LPO is not expressed (data not shown). Our autoimmune thyroid disease (AITD) serum pool does not recognize bLPO, however, there are some data showing cross-reaction between bLPO and some AITD serum samples [39].

The link between thyroid dysfunction and breast cancer development has been intensively studied for many years, however with conflicting results. There are studies showing a positive correlation between hyperthyroidism, characterized by elevated levels of thyroid hormones, and breast cancer risk [40–42], whereas others found a protective role of hypothyroidism in breast cancer development [43]. In contrast, increased risk of breast cancer has been reported in post-menopausal women in the hypothyroid state [44]. Moreover, no association between thyroid function and breast cancer has been observed [45–48]. A recently reported nationwide Danish cohort study restricted to women with hypo- and hyperthyroidism has clearly shown that the hormonal status of the thyroid gland influences the breast cancer risk [49]. Women with hyperthyroidism were more prone to develop breast cancer, whereas hypothyroidism was rather a protecting factor [49]. The observed association between thyroid disorders and breast cancer risk may be explained by various biological mechanisms. Noteworthy, there are data showing that thyroid hormones are involved in breast cancer development and growth. The expression of thyroid hormone receptors is detected in normal and tumoral breast tissues, nevertheless their role in the breast cancer etiology and progression is poorly understood [50]. Recent data also show that triiodothyronine can stimulate estrogen receptor in breast cancer-derived cell lines increasing their proliferative activity [51]. Autoimmune thyroid disorders are predominantly associated with the presence of thyroid autoantibodies directed to TPO and thyroglobulin (reviewed in [4, 17]). In our study, 12 out of 56 (21.4%) of breast cancer patients were positive for TPOAbs whose levels ranged from low to high. Similar frequency of TPOAbs occurrence in breast cancer patients has been also reported by others [8, 12, 14]. Performed analyses, however, have not shown statistically significant difference in TPO mRNA expression level between patients with and without TPOAbs. There was also no association between TPOAbs positivity and TNM classification or histological grading. Similar results were obtained by Farahati and coauthors on the larger group of TPOAbs positive breast cancer patients [15]. As already mentioned, the role of TPOAbs in breast cancer progression and prognosis has been suggested, however, the biological mechanism remains unclear. Some insights into the potential role of these antibodies in cancer patients may be provided by AITD mechanism analyses. In thyroid autoimmunity, TPOAbs have been shown to be implicated in thyrocyte damage through the activation of the complement cascade [52] and antibody-dependent cytotoxicity mechanism [32]. Moreover, these autoantibodies may participate in the antigen presentation to autoaggressive T cells and intensify the autoimmunity aggression process [53].

## Conclusions

We have demonstrated that TPO is present in both cancer and normal breast tissue, which confirms the results of Muller and collaborators [21]. Our data reveal that TPO in breast tissue is recognized by IDR-A and IDR-B-specific monoclonal antibodies and TPOAbs from patients with AITD. Thus, we show for the first time that TPO expressed in breast cancer tissue shares immunological characteristics with the thyroid-expressed TPO. Moreover, we detected that TPO transcript levels are lower in more advanced breast cancers. However, further studies are necessary to fully understand the mechanisms by which TPOAbs may have a protective effect against breast cancer metastases.

## Supporting information

S1 Fig Detection of bovine lactoperoxidase (bLPO) with antibodies against thyroid peroxidase (TPO) (ab76935, mAb A4, mAb 47) by Western blot. The 10376-1-AP anti-LPO antibody was used as a control. (A-C) Anti-TPO antibodies were preabsorbed (P) with the excess of purified human TPO. (D) Preabsorption (P) of anti-LPO Ab with bLPO (P with bLPO) and with hTPO (P with hTPO). 100 ng of hTPO or bLPO was loaded. NP, non-preabsorbed Ab.
(TIF)

S2 Fig Specific control for pooled human serum from autoimmune thyroid disease (AITD) patients with high titer of autoantibodies against thyroid peroxidase (TPOAbs). (A) Immunoprecipitation of human TPO (hTPO) using a pool of sera negative for TPOAbs (lane 2) and sera with high levels of TPOAbs (lane 3). 50 ng of hTPO was loaded in lane 1. (B) Immunoprecipitation of bovine lactoperoxidase (bLPO) using TPOAb-negative (lane 2) and TPOAb-positive (lane 3) patient sera pools. 50 ng of bLPO was loaded in lane 1. The signals were visualized using TPO-specific monoclonal antibody A4 (A) and LPO-specific 10376-1-AP antibody (B).
(TIF)
