# Supplementary figures and images for: Thyroid peroxidase (TPO) expressed in thyroid and breast tissues shows similar antigenic properties
Source: PLoS One. 2017 Jun 2;12(6):e0179066. doi: 10.1371/journal.pone.0179066 (PMC5456382; doi:10.1371/journal.pone.0179066)

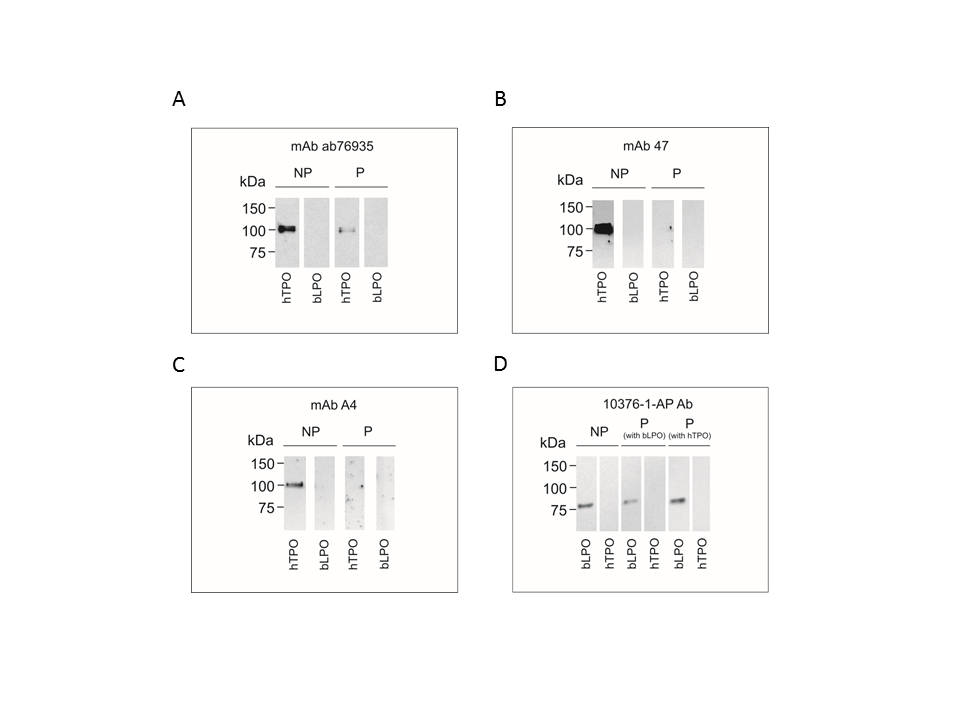

Supplement: S1 Fig — The 10376-1-AP anti-LPO antibody was used as a control. (A-C) Anti-TPO antibodies were preabsorbed (P) with the excess of purified human TPO. (D) Preabsorption (P) of anti-LPO Ab with bLPO (P with bLPO) and with hTPO (P with hTPO). 100 ng of hTPO or bLPO was loaded. NP, non-preabsorbed Ab. (TIF) [file pone.0179066.s001.tif]

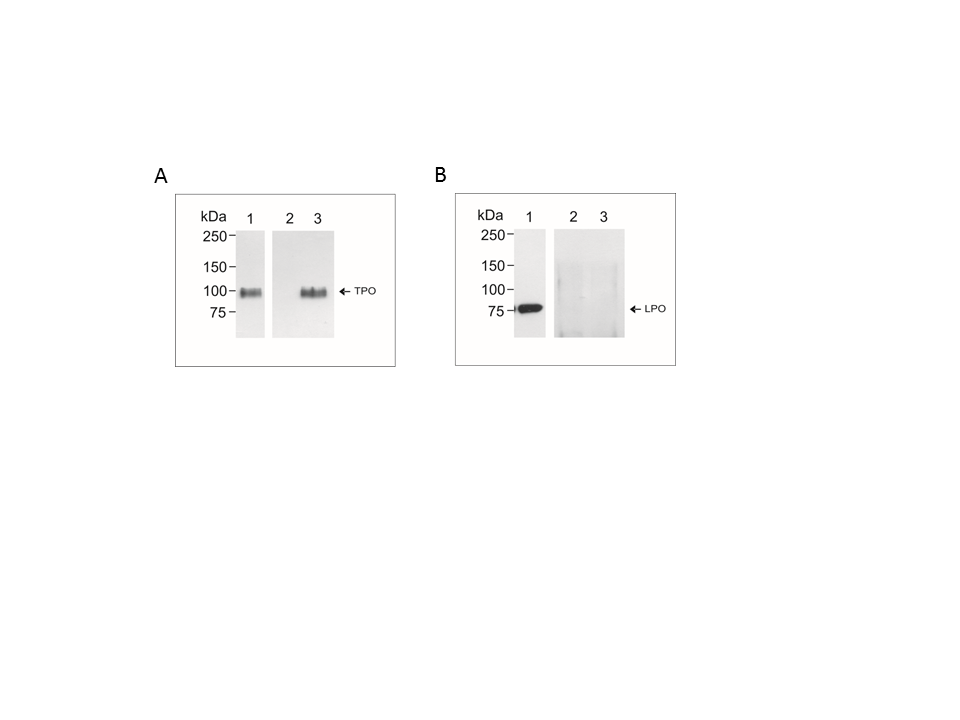

Supplement: S2 Fig — (A) Immunoprecipitation of human TPO (hTPO) using a pool of sera negative for TPOAbs (lane 2) and sera with high levels of TPOAbs (lane 3). 50 ng of hTPO was loaded in lane 1. (B) Immunoprecipitation of bovine lactoperoxidase (bLPO) using TPOAb-negative (lane 2) and TPOAb-positive (lane 3) patient sera pools. 50 ng of bLPO was loaded in lane 1. The signals were visualized using TPO-specific monoclonal antibody A4 (A) and LPO-specific 10376-1-AP antibody (B). (TIF) [file pone.0179066.s002.tif]
